# Always Approach the Bright Side of Life: A General Positivity Training Reduces Stress Reactions in Vulnerable Individuals

**DOI:** 10.1007/s10608-015-9716-2

**Published:** 2015-09-25

**Authors:** Eni S. Becker, Hannah Ferentzi, Gina Ferrari, Martin Möbius, Suzanne Brugman, José Custers, Naline Geurtzen, Joelle Wouters, Mike Rinck

**Affiliations:** Behavioural Science Institute, Radboud University Nijmegen, PO Box 9104, 6500 HE Nijmegen, The Netherlands

**Keywords:** Approach-avoidance, Positivity training, Emotional vulnerability

## Abstract

Emotional disorders are characterized by cognitive biases towards negative stimuli, and a lack of biases towards positive ones. Therefore, we developed a cognitive bias modification training, modifying approach-avoidance tendencies to diverse emotional pictures. In Study 1, a negative training (pull negative, push positive pictures) was compared to a positive training (vice versa) in 141 students. The pre-existing positivity bias remained after positive training, but reversed into a negativity bias after negative training. This effect transferred to an attentional bias. The training affected neither mood nor emotional vulnerability to stress. In Study 2, we investigated the effects of the positive training in 102 dysphoric and non-dysphoric students, all in a sad mood state. Compared to placebo training, the positive training strengthened a positivity bias, and it reduced emotional vulnerability in dysphoric students. This suggests potential therapeutic value of the training, but further studies are needed.

## Introduction

According to cognitive theories, the development and maintenance of emotional disorders such as depression or anxiety can be partly attributed to selective processing of emotionally relevant information, also referred to as cognitive biases (e.g., Beck and Clark 1997; Mathews and MacLeod 2005; Rapee and Heimberg 1997). Individuals suffering from emotional disorders and those with a higher vulnerability (MacLeod and Mathews 2012) selectively attend to negative or potentially threatening information, they remember it better, and they interpret ambiguous stimuli in a more negative or dysfunctional manner than healthy individuals do (see Hertel and Mathews 2011; Mathews and MacLeod 2005). In addition to these biases towards negative materials, a lack of positive biases also characterizes many emotional disorders (e.g., Liang et al. 2011), whereas healthy individuals preferentially process positive information (e.g., Deldin et al. 2001; Dunn et al. 2007; Joormann and Gotlib 2007). This positivity bias plays an important role in mood regulation (e.g., Isaacowitz et al. 2009; Joormann et al. 2007; Xing and Isaacowitz 2006). Thus, it seems that both the presence of negative biases and the absence of positive biases are characteristic of emotional disorders.

In order to identify whether cognitive biases are more than mere symptoms of the emotional disorders, researchers developed so-called cognitive bias modification (CBM) paradigms. These are various computerized training methods that can be used to experimentally manipulate cognitive biases in attention, memory, interpretation, or approach-avoidance tendencies. Studies making use of CBM paradigms provided increasing evidence for the causal role of cognitive biases in emotional vulnerability and dysfunction (Clarke et al. 2008; MacLeod et al. 2002; See et al. 2009). Moreover, CBM procedures may also be of therapeutic value in clinical settings. A growing body of literature shows that various forms of extended CBM procedures can effectively reduce clinical symptoms (for a meta-analysis, see Hallion and Ruscio 2011), for instance in generalized anxiety disorder (GAD; for a meta-analysis see, Hakamata et al. 2010), social anxiety disorder (e.g., Beard and Amir 2008; see Beard et al. 2012, for a review), generalized social phobia (Amir et al. 2009), and depression (Wells and Beevers 2010). Also, CBM procedures can help to decrease relapse rates in alcoholics (Wiers et al. 2011; Eberl et al. 2013).

While most CBM studies focused on the experimental manipulation of negativity biases, only a few CBM studies have experimentally addressed the protective role of positive information processing in emotional vulnerability. Johnson (2009) explicitly instructed participants to selectively attend to happy faces and to avoid angry faces during a dot-probe task. Compared to a group which received no instructions, participants who attended to positive faces reported less frustration in response to a subsequent stressor. Similarly, Taylor et al. (2011) showed that greater shifts in attention towards positive, social-evaluative word stimuli following attention training were related to attenuated anxiety reactivity.

Furthermore, most of the CBM studies so far made use of stimulus materials that were highly disorder-specific, such as social-evaluative words or faces expressing disgust in social anxiety (e.g., Taylor et al. 2011). However, there is at least one study showing that in healthy children (Broeren and Lester 2013), a bias towards positive materials is not restricted to specific content. Instead, it extends to all positive information that is relevant to the child, suggesting that the positivity bias in healthy individuals reflects a general orientation. It is conceivable that in healthy adults, the positivity bias is also of a more general nature. Thus, it might be important to focus on the processing of diverse categories of positive information instead of content-specific stimuli, when aiming to facilitate a positivity bias in emotionally disordered patients.

This view is supported by new developments in treatment, which suggest to focus on the commonalities in biased information processing between different emotional disorders and on the development of unified treatment protocols, in order to facilitate treatment efficiency and efficacy (Moses and Barlow 2006), which is also supported by the tripartite model (Clark and Watson 1991), in which a general negative affectivity component is assumed to be a higher-order factor for both anxiety and depression, whereas anxiety and depression differ in symptomatology only at a ‘lower’ level. Moses and Barlow (2006) also suggest that a unified treatment approach for emotional disorders should contain the modification of emotion-driven behaviors (so-called action tendencies). According to approach-avoidance models (e.g., Elliot 2006), positive stimuli elicit approach motivations and subsequently approach behavior, while negative stimuli elicit avoidance motivation and avoidance behavior. In emotional disorders, these natural tendencies are frequently compromised. For instance, individuals with depressed symptoms show low reward sensitivity, which is associated with lower approach behavior towards rewarding cues. At the same time, these individuals show difficulties in disengaging from negative stimuli, reflected in a range of biases promoting the processing of negative information (see Trew 2011). Consequently, they might profit from a training that simultaneously promotes approach towards positive cues and avoidance from negative cues.

Action tendencies can be efficiently measured in experimental settings by using an approach-avoidance task (AAT, Rinck and Becker 2007), and there are a number of studies that examined these approach-avoidance tendencies in patient samples (e.g., Heuer et al. 2007; Lange et al. 2008; Reinecke et al. 2012; Schuck et al. 2012). Most interestingly, the approach-avoidance task has also been used successfully for cognitive bias *modification*: Patients were trained to use a joystick to pull pictures of positive, healthy stimuli closer, and push pictures of unhealthy, negative pictures away. For instance, an alcohol-avoidance training for alcohol-dependent patients reduced relapse rates significantly (Eberl et al. 2013, 2014; Wiers et al. 2011). Moreover, there are promising results regarding the training to approach faces in social anxiety (Rinck et al. 2013; Taylor and Amir 2012). Therefore, the training of approach-avoidance tendencies might be a good starting point, especially with the goal of a more unified treatment approach. It is important to note that this training differs from most previous ones in that it does not train attentional vigilance or avoidance. In fact, in the AAT, single pictures are presented, and the participant has to attend to each one before pulling it closer (approach) or pushing it away (avoidance). Thus, attention is held constantly high, while action tendencies are selectively trained.

To sum up, most CBM studies focused on the elimination of biases towards content-specific negative cues. There is a lack of CBM techniques designed to promote a healthier bias towards non-specific positive information and simultaneously eliminate an unspecific negativity bias. The lack of a positivity bias is associated with difficulties in emotion regulation. Exploring the promotion of a general positivity bias by means of CBM training techniques, rather than focusing on the isolated reduction of disorder-specific negative biases, is an interesting new approach with high theoretical impact. Above that, CBM that fosters a broad positive bias might be a powerful cognitive intervention with high efficiency, which will add to the development of treatments and prevention programs of emotional disorders.

Following this line of reasoning, we developed a CBM procedure which includes diverse categories of positive and negative pictures (e.g., objects, animals, humans), in order to train general, disorder-nonspecific action tendencies. Consequently, the emotional stimuli do not apply to any disorder in particular. In the positive training condition, the training strengthens the approach of positive stimuli and simultaneously the avoidance of negative stimuli. Both contingencies (approach positive, avoid negative) foster a differential reaction pattern based on stimulus valence across a broad range of categories. During this *general approach*-*positive*-*avoid*-*negative training* (in short: *positivity training*), participants have to react to pictures on a computer screen by pulling or pushing a joystick. Depending on the joystick movement, the pictures increase or decrease in size, creating an approach or avoidance impression, respectively.

In the first study, we investigated whether such a general training is indeed able to modify pre-existing approach-avoidance tendencies, and if the modification generalizes to other cognitive processes (i.e., attention). Moreover, we examined whether this training affects emotional vulnerability. Given the proof-of-principle nature of this first study, we did not include a neutral control condition. Instead, we compared an approach-positive-avoid-negative training to the opposite approach-negative-avoid-positive training. In the second study, we followed a more clinical approach by investigating the effects of the general positivity training in a sample of dysphoric and non-dysphoric students.

## Study 1

The aim of Study 1 was to investigate whether a general approach-avoidance training with diverse emotional pictures is able to modify action tendencies, whether the effect generalizes to attentional biases, and if the training affects emotional reactivity to a stressor. To this end, participants received a general approach-avoidance training, either towards positive and away from negative pictures (PT: positivity training) or towards negative and away from positive pictures (NT: negativity training). These two groups were then compared with regard to changes in approach-avoidance tendencies. Participants receiving the PT were expected to become faster at pushing negative pictures and pulling positive pictures than vice versa (indicative of a positivity bias), whereas participants receiving the NT were expected to become faster at pulling negative pictures and pushing positive pictures than vice versa (indicative of a negativity bias).

It has been suggested that approach or avoidance movements influence the motivational orientation and subsequently enhance the processing of positive or negative stimuli (positive–approach, negative–avoidance; Neumann and Strack 2000). To examine such putative crossover effects of the modified approach-avoidance tendencies on attentional processes, participants’ attentional bias was measured by means of a dot-probe task after the training. We expected that the training effects would generalize to an attentional bias, with the NT group attending more to negative stimuli and the PT group more to positive stimuli.

In line with earlier research on the relationship between cognitive biases and emotional vulnerability, it was finally expected that, compared to the negativity training, the positivity training would result in attenuated stress-reactivity to a subsequent stressful task. Based on previous findings of, for instance, MacLeod et al. (2002), we did not expect a direct effect of the training on mood.

### Methods

#### Participants

Participants of this study were 141 first-year psychology and educational science students of Radboud University Nijmegen, the Netherlands. They were randomly assigned to either the PT group or the NT group. The groups did not differ in age (PT: *M* = 20.46, *SD* = 2.86; NT: *M* = 20.61, *SD* = 2.63; *t*(139*)* = .32, *p* = .749), gender (PT: 11 males, 59 females; NT = 8 males, 63 females; χ^2^(1, 141) = .60, *p* = .439), or mood before the experiment (PT: *M* = 5.89, *SD* = 4.31; NT: *M* = 6.10, *SD* = 3.99; *t*(139) = .30, *p* = .642). Participants received course credit in return for their participation.

#### Materials

##### Mood Scales

Participants had to indicate their current mood state at four different time points during the experiment. For this purpose, statements were presented on a computer screen and participants had to indicate on a six-point Likert scale to what extent they agreed or disagreed with it. Three of these statements (happiness, sadness, relief) reflected a depression-related dimension. They were included to check whether the training had any undesired and immediate negative effects on mood. The other three statements (tension, relaxation, anxiety) reflected a stress-related mood dimension. They were included to measure effects of the anagram stress task on stress vulnerability.

##### Emotional Pictures

A set of 100 positive and 100 negative pictures, representing a broad range of different categories (e.g., animals, humans, objects) were selected from several sources, including the International Affective Picture System (IAPS; Lang et al. 2005) and the Karolinska Directed Emotional Faces database (KDEF; Lundqvist et al. 1998). Positive and negative pictures were of equivalent emotional intensity, and they were selected to be gender-non-specific. The pictures were selected based on the ratings supplied by the authors of the IAPS and the KDEF. Highly arousing pictures (e.g., mutilations, sexual activities) were excluded. Two equivalent sets of 50 positive and 50 negative pictures each were created. For each participant, only one of the two sets was used in the training AAT, and we counterbalanced across participants which set was used during the training. Afterwards, all pictures were used in the dot-probe task to allow for a test of generalization to untrained pictures.

For the AAT, each picture existed in a slightly left-tilted (5°) and in a slightly right-tilted (5°) version. Moreover, to allow for the zooming effect of the AAT, seven different sizes of each picture version were created, with the largest picture filling the full height of the computer screen (768 pixels), and the smallest picture being approx. 90 pixels high (as described in Rinck and Becker 2007). Depending on the orientation of the picture (landscape or portrait), the width varied between 70 pixels (smallest portrait) and 1024 pixels (largest landscape). Corners and edges of the pictures were blurred in order to prevent participants from responding by looking at the pictures’ corners only.

#### Experimental Tasks

##### Approach-Avoidance Task (AAT)

During the whole AAT, the participants’ task was to respond to the pictures by pulling or pushing a joystick that was securely positioned in the middle of the table in front of the computer screen. The participants initiated each trial by holding the joystick in a central position and pressing a button of the joystick with the index finger, upon which a picture of medium size appeared on the screen. Participants were instructed to respond as quickly as possible to the tilt of each picture, by pulling left-tilted pictures closer and pushing right-tilted ones away with the joystick. To create the visual impression that the picture itself was pushed away or pulled closer, the picture changed in size dynamically with every joystick movement. As soon as the joystick was moved by all the way into the correct direction, the picture disappeared and the trial ended with a black screen. If the joystick was moved into the incorrect direction, the picture did not disappear. Participants then had to correct their movement in order to be able to proceed to the following trial. Response accuracy therefore was 100 % for all trials. The computer automatically recorded participants’ movements and reaction times (RTs), that is, the time from appearance of each picture to its disappearance.

The training in this study was divided into five phases. The first phase consisted of 10 practice trials. This was followed by an assessment AAT that consisted of 40 trials, in which both positive and negative pictures had to be both pulled and pushed equally often. Immediately after this assessment and unbeknown to the participants, the training AAT followed, consisting of 380 trials. In this phase, participants in the positive training group (PT) had to pull all positive pictures closer and to push all negative ones away. For participants in negative training group (NT), the contingencies were reversed. Thereafter and again unbeknown to participants, the training AAT changed into a post-assessment AAT that was identical to the pre-assessment. Since we did not aim to investigate generalization of training effects within the training itself, all pictures presented in the pre- and post-assessment were also used during training. Finally, after the dot probe task (see below), a booster-training block of 100 trials was administered to ensure that training effects were not reduced by the dot probe task. The whole joystick task took approximately 25 min, with three short breaks during the training phase.

##### Dot-Probe Task

Each dot-probe trial commenced with a 500 ms fixation cross in the center of the screen. Afterwards, a positive and a negative picture appeared above and below the center of the screen. The position of the pictures was randomized, such that each picture appeared in the upper or lower screen location with equal probability. The pictures were presented for 500 ms, after which an arrow (pointing left or right) appeared at the position of one of the pictures. Participants were asked to react to the arrow as quickly and correctly as possible by pressing either a left or a right button on the keyboard, depending on whether the arrow pointed to the left or to the right. Upon the participant’s response, the screen was cleared and followed by a new trial.

The whole task consisted of 10 practice trials and 100 assessment trials. On half of the trials, the probe replaced the positive picture and on the other half, it replaced the negative picture. In order to test generalization effects of the training on attention bias, 50 picture pairs were included that consisted of the pictures previously used in the AAT (trained pictures), while the other 50 picture pairs consisted of new pictures (untrained pictures). Trained and untrained picture pairs were presented in random order.

##### Anagram-Stress Task

To elicit stress in the participants, an adapted version of the anagram-stress task by MacLeod et al. (2002) was used. Twenty letter strings were constructed for this task. Seven of these anagrams were solvable (2 easy, 5 difficult) in that the letters within each string could be rearranged to spell a word. Thirteen of the anagrams were unsolvable, in that the letter strings could not be rearranged to form any word. However, all anagrams were described as being solvable. As some participants were of German origin, a German version and a Dutch version of the task were created. Participants were instructed to solve as many anagrams as possible in a period of 5 min. The anagrams were presented on a sheet of paper, and participants were free to skip anagrams they could not solve. After 5 min, the experimenter returned to the room and indicated, independently of the real performance, that the participant’s performance level was unusually low.

#### Procedure

Participants were tested individually. After providing informed consent, participants were randomly assigned to either the PT group or the NT group, after which they filled in both mood scales and completed the AAT. Next, the mood scales were presented for the second time. Then, participants completed the dot-probe task followed by the AAT booster-training, to make sure that the dot-probe task did not reduce AAT training effects. After that, participants had to fill in the mood scales for the third time. In order to assess effects of the AAT training on emotional vulnerability, participants subsequently completed the anagram stress task, and filled in the mood scales for the last time. At the end of the session, participants filled in an awareness check questionnaire and were paid. All participants were debriefed per e-mail after the end of data collection.

### Results

#### AAT Training Effect

To evaluate changes in the approach-avoidance tendencies between the two assessment phases (i.e., before versus after the training AAT), median RTs were calculated for the 4 combinations of pushing versus pulling and positive versus negative pictures. Hereby, the fastest and the slowest 1 % of all RTs were excluded to reduce the potential effect of outliers. As the response accuracy of this task is necessarily 100 % as explained above, there were no RTs of incorrect responses to be excluded. A compatibility score was computed separately for each participant and each assessment phase (pre and post) by subtracting median RTs of compatible trials (i.e., pull positive pictures, push negative pictures) from median RTs of incompatible trials (i.e., push positive pictures, pull negative pictures). Positive compatibility scores reflect faster reactions on compatible trials (i.e., a positivity bias).

The compatibility effects were then subjected to a 2 (within-subjects factor *time*: pretest vs. posttest) × 2 (between-subjects factor *group:* PT vs. NT) repeated measures analysis. This analysis revealed the expected significant interaction, *F*(1, 139) = 36.77, *p* < .001, η_p_^2^ = .21. Separate analyses for each group indicated that only the NT group showed the expected training effect, *F*(1, 70) = 61.33, *p* < .001, η_p_^2^ = .47: In line with their training condition, they reacted faster on compatible trials than on incompatible trials before the training, and faster on incompatible trials than on compatible trials after the training. This effect was not found for the PT group, *F*(1, 69) = .39, *p* > .5, η_p_^2^ = .01; their pre-existing positive compatibility effect remained unchanged (see Table 1 for means and SDs).

**Table 1:** Mean reaction times (standard deviations) in milliseconds of the Approach-Avoidance Training and the resulting compatibility effects in Study 1

	Positive training		Negative training	
	Pre-training (T1)	Post-training (T2)	Pre-training (T1)	Post-training (T2)
Pull negative	834 (191)	828 (175)	852 (196)	783 (146)
Push negative	782 (199)	731 (145)	808 (167)	829 (179)
Pull positive	783 (168)	800 (158)	792 (163)	842 (165)
Push positive	826 (175)	816 (168)	844 (147)	765 (120)
Compatibility effect	96 (170)	112 (193)	96 (166)	−123 (154)

#### Dot-Probe Task

To analyze crossover effects from the AAT to the dot-probe task, we again computed a bias score. Only correct trials of the dot-probe task were taken into the analyses, after excluding the fastest 1 % and the slowest 1 % of all remaining reaction times. Of these trials, median RTs were calculated per participant, separately for each of the four combinations of picture type (trained vs. untrained) and probe location (positive vs. negative picture). These median RTs were subjected to a 2 (between-subjects factor *Group*: PT vs. NT) × 2 (within-subjects factor *picture type*: trained vs. untrained) × 2 (within-subjects factor *probe location*: positive picture vs. negative picture) repeated-measures ANOVA.

The analysis revealed a significant two-way interaction between probe location and group, *F*(1, 139) = 8.11, *p* = .005, η_p_^2^ = .06, indicating the expected crossover effect from the AAT training to the dot-probe task. Adding the initial compatibility effect as a covariate to the analysis revealed that this interaction effect was moderated by the compatibility scores, *F*(1, 137) = 4.23, *p* = .041, η_p_^2^ = .03. This suggests that the effect of approach-avoidance training on attention was more pronounced in participants with an initially low compatibility effect. Separate analyses for the two groups showed that the PT group reacted more quickly when the probe replaced the positive picture (*M* = 595, *SD* = 42) than when it replaced the negative one (*M* = 602, *SD* = 44), *F*(1, 69) = 5.74, *p* = .019, η_p_^2^ = .08. In the NT group, RTs for positive pictures (*M* = 600, *SD* = 54) and negative pictures (*M* = 596, *SD* = 55) did not differ significantly, *F*(1, 70) = 2.7, *p* > .10, η_p_^2^ = .04. The probe location by group interaction was marginally significant for both untrained pictures, *F*(1, 139) = 3.86, *p* = .051, η_p_^2^ = .03, and trained pictures, *F*(1, 139) = 3.70, *p* = .056, η_p_^2^ = .03, pointing to a generalization from trained to untrained pictures. Because of the similar effects observed for trained and untrained pictures, the three-way interaction of probe location, picture type, and group was not significant, *F*(1, 139) = .10, *p* > .7, η_p_^2^ = .001.

#### Mood and Stress After Training

To analyze immediate effects of the training on mood, a multivariate analysis of covariance (MANCOVA) was conducted to compare the two groups’ mood state after the training, with the three mood ratings (happy, sad, relieved) as dependent variable, and group as independent variable (see Table 2). Mood ratings before the training served as covariates. A corresponding analysis was conducted on the three stress ratings (tense, anxious, relaxed). The MANCOVA was favored over a repeated-measures ANOVA because the stress scales showed violations of sphericity (Stevens 2002). The analyses revealed neither an immediate training effect on the mood scales nor on the stress scales (*p* > .398). Including the baseline compatibility scores as a covariate resulted in a marginally significant interaction effect of baseline compatibility scores with training on mood, *F*(3, 132) = 2.46, *p* = .066, η_p_^2^ = .05, which was mainly caused by the happiness subscale, *F*(1, 134) = 2.16, *p* = .083, η_p_^2^ = .02. This suggests that stronger compatibility scores were related to higher happiness after the training.

**Table 2:** Mean scores (standard deviations) of mood and stress scores before (T1) and after (T2) the training and before (T3) and after (T4) the stress task in Study 1

	Positive training				Negative training			
	T1	T2	T3	T4	T1	T2	T3	T4
Mood								
Happy	4.13	3.83	3.71	3.5	4.13	3.66	3.58	3.41
	(1.05)	(1.09)	(1.17)	(1.16)	(1.1)	(1.21)	(1.17)	(1.21)
Sad	1.71	1.73	1.64	1.77	1.56	1.73	1.63	1.73
	(1.17)	(.98)	(.95)	(1.07)	(1.04)	(1.03)	(1)	(1.15)
Relieved	2.91	3.06	3.2	3.07	2.72	2.68	2.76	2.69
	(1.07)	(1.06)	(1.11)	(1.15)	(1.61)	(1.23)	(1.21)	(1.27)
Stress								
Tense	2.44	2.33	2.11	2.26	2	2	1.87	2.35
	(1.34)	(1.27)	(1.08)	(1.33)	(1.32)	(1.7)	(1.04)	(1.28)
Anxious	1.39	1.5	1.46	1.44	1.32	1.44	1.31	1.37
	(.8)	(.81)	(.79)	(.9)	(.82)	(.81)	(.73)	(.76)
Relaxed	4.1	3.9	3.94	3.79	4.14	3.82	4.14	3.93
	(1.26)	(1.19)	(1.41)	(1.36)	(1.42)	(1.27)	(1.25)	(1.29)

#### Mood and Stress After Anagram Task

To assess effects of the training on emotional reactivity to the anagram-stress task, MANCOVAs were conducted for the three stress ratings and the three mood ratings, with the same scales prior to the task serving as covariates (see Table 2). The MANCOVAs of the stress scale and the mood scale after the anagram-stress task indicated no training effect either (*p* > .3).^1^

### Discussion

The results of Study 1 indicate that it is possible to modify action tendencies by means of a general approach-avoidance training. Specifically, the NT was able to reverse a positive compatibility effect (i.e., positivity bias) into a negative one: Participants of the NT group became faster to approach negative pictures and to avoid positive pictures. This finding suggests that CBM training effects are not restricted to the use of specific, disorder-relevant stimuli. Instead, they extend to the processing of unspecific, emotionally valenced information in general. However, the positivity training was not effective in increasing an existing positivity bias even further. This might be attributable to the pre-existing compatibility effect: Both groups showed a positivity bias before the training, indicative of faster approach reactions to positive and faster avoidance reactions to negative stimuli. In terms of a ceiling effect, strengthening an already existing bias is more difficult than altering a bias by means of training (i.e., reversing a positive bias into a negative one). It therefore remains to be established if a positivity bias can be induced in individuals who lack such a positive bias, or if a negativity bias can be reversed by means of the positivity training.

Furthermore, a crossover effect of the AAT on the dot-probe task was found: After the training, the groups differed in their attention allocation to positive and negative stimuli, such that attention was biased towards the class of previously approached stimuli. These results support the assumption that not only perceptions automatically trigger a motivational orientation and corresponding action tendencies, but that there is also a causal effect in the opposite direction: Approach-avoidance movements may influence the motivational orientation and subsequently enhance the processing of approached stimuli (Neumann and Strack 2000). Importantly, these results also indicate that the approach-avoidance training did not merely work at the stimulus–response level: Participants learned to connect a kind of movement (e.g., pull) with a whole class of stimuli (e.g., positive pictures). This suggests a general influence of the AAT on the processing of emotionally valenced information.

Subsequent analyses revealed that the PT group showed an attentional bias towards positive pictures after the training, whereas no bias was found in the NT group. As described above, the AAT reversed the pre-existing, positive approach-avoidance bias in the NT group, which might explain the lack of a positive attentional bias in this group. This is supported by the fact that the PT group showed a positive attentional bias, as typically found in healthy participants (Joormann and Gotlib 2007). Unfortunately, we did not measure the attentional bias prior to the AAT. Inferences about changes in bias from pre- to post-training are thus based on assumptions that cannot be tested with the data of the current study. Clearly, future studies should include both pre- and post-measures of attentional bias.

Regarding our second research question, we found that there were no direct effects of the AAT on mood. This suggests that the training procedures did not serve as a mood induction. This finding is in line with the existing literature, which suggests that CBM training effects become apparent only when participants are subsequently exposed to a stressful situation (for a review see, Hallion and Ruscio 2011). However, we did not find such previously reported, attenuating effects on emotional vulnerability either. The trainings did not differentially affect stress reactivity of participants in response to the subsequent anagram task. This suggests that the general approach-avoidance training might not have an impact on emotional vulnerability. An alternative explanation for the absence of an effect of training on emotional vulnerability might be that the stress task used in this experiment was not adequate for inducing sufficiently high stress levels. This is also reflected in the generally low stress scores observed. In order to facilitate differences in stress reactivity between the groups, the task might need to be adapted in order to provoke stronger stress experiences.

## Study 2

Study 1 showed that a general approach-avoidance training is effective in modifying pre-existing action tendencies in healthy individuals and that this effect transfers to attentional processes. In line with previous CBM studies, the AAT was effective in training individuals towards negative stimuli. However, we were not able to induce a bias towards positive stimuli, as the participants already had a pre-existing positivity bias. As a result of this ceiling effect, the initial positivity bias remained unchanged in the positivity-training group.

Therefore, the aim of Study 2 was to investigate whether the general approach-avoidance training is able to induce a positivity bias in individuals who are assumed to lack such a positivity bias. In this study, we investigated the training in dysphoric and non-dysphoric students. All participants were brought into a sad mood state, because cognitive biases are influenced by state effects and seem to come to play even more clearly as a consequence of depressive trait factors (Mathews and MacLeod 2005). Above that, we tested whether the positivity training affects stress reactivity. Because of the additional time needed for the mood induction, we had to drop the dot-probe task from this study.

To prevent a potential ceiling effect like the one observed in Study 1, a negative mood state (i.e., analogue depression) was induced prior to the training by means of a sad movie. Research has shown that such mood inductions may be sufficient to affect cognitive processes (e.g., attention bias: Bradley et al. 1997; memory bias: Fitzgerald et al. 2011; Matt et al. 1992). A negative mood induction re-activates latent depressogenic cognitive structures in emotionally vulnerable individuals (e.g., Beck 1967). Hence, it also serves to elicit a negativity bias in the dysphoric group (for a review on this procedure see Scher et al. 2005). Considering the importance of positive biases in emotion regulation, we argue that emotionally vulnerable individuals (dysphoric students) should particularly benefit from the training.

Study 2 focused on reducing stress reactivity by means of the general positivity training. Participants were either trained towards positive stimuli and away from negative ones (PT group), or they received a sham training (control group), in which they pulled and pushed positive and negative pictures equally often. For ethical reasons, the study did not include training towards negative stimuli. Again, direct effects of the training on mood as well as indirect effects on mood in response to a laboratory stressor were investigated.

In order to address methodological issues discussed with regard to Study 1, the anagram-stress task in Study 2 was improved. Specifically, we increased time pressure and we made the task more credible to participants, in the sense that anagrams seemed solvable at first glance. Moreover, the mood rating “relieved” was replaced by “content”, as we considered this item to better reflect a positive mood state.

We expected that (1) compared to the sham training, a bias towards positive stimuli can be induced or strengthened by means of the general positivity training in both dysphoric and non-dysphoric individuals who are in a sad mood state. Based on the discussed literature (e.g., Taylor et al. 2011), we expected that (2) the induced positivity bias has a buffer function, in that it decreases participants’ negative affective response to a stressful situation. In order to investigate the therapeutic relevance of the training, we differentiated between dysphoric and non-dysphoric students.

### Methods

#### Participants

103 Dutch (*n* = 63) and German (*n* = 40) first-year psychology and educational science students of Radboud University Nijmegen participated in this study in return for course credit. Participants were randomly assigned to either the training group or the control group. As this study was designed to address individuals who are in a sad mood, participants who did not respond to the negative mood induction were excluded from further analyses (dysphoric: *n* = 13; non-dysphoric: *n* = 29). This resulted in a remaining sample size of *n* = 61.

A major aim of the study was to assess the therapeutic value of the general positivity training for dysphoric individuals. Consequently, the sample was split into a dysphoric and a non-dysphoric group, based on their scores on the Self-Rating Depression Scale (SDS;see below). Following Zung (1973), participants with a sum score of 40 or lower were classified as non-dysphoric (*n* = 36), whereas those scoring higher than 40 were classified as dysphoric (*n* = 25). The resulting four groups (i.e., dysphoric-training vs. dysphoric-control, non-dysphoric-training vs. non-dysphoric control) did not differ in size, mean age, gender distribution, or nationality distribution. Moreover, the two dysphoric groups (training versus control) had comparable pre-experimental SDS scores (training: *M* = 46.3, *SD* = 3.9; control: *M* = 47.7, *SD* = 5.3), as had the two non-dysphoric groups (training: *M* = 35.9, *SD* = 3.1; control: *M* = 34.6, *SD* = 4.6).

#### Materials

##### Mood Measurements

To assess the presence and magnitude of depressive symptoms in the participants, a Dutch computerized version of the Self-Rating Depression Scale (SDS; Zung 1965, 1973) was administered, which has been shown to have good psychometric properties (Peeters et al. 1993). As suggested by Zung (1973), a score above 40 is used as an indication of mild, clinically significant depression (see also Bitsika et al. 2010).

To investigate changes in mood, participants rated their feelings on six mood ratings on a 7-point Likert scale, ranging from 0 (*totally disagree*) to 6 (*totally agree*). These six mood ratings can be categorized into a depressed mood dimension and a stress-related mood dimension. The depressed mood dimension consisted of the ratings happiness, sadness, and contentment. These were used as indicators for a depressive mood state and served as manipulation check of the mood induction, as well as dependent variables for the first hypothesis. The stress-related mood dimension consisted of the ratings tension, relaxation, and anxiety. These functioned as indicators of the current stress level of participants and served as dependent variables for the second hypothesis.

##### Emotional Pictures

For the approach-avoidance task, the same set of stimuli as in Study 1 was used. However, all 100 positive and 100 negative pictures were presented to each participant during the training.

#### Experimental Tasks

##### Mood Induction

To induce a negative mood state, three sequences of a sad movie (“Sophie’s Choice”) were shown to the participants on a computer screen. These sequences have repeatedly been effective in eliciting negative mood (e.g., Randall and Cox 2001). All three sequences together lasted about 20 min. Before and after each sequence, participants were asked to report their current emotion on an 11-point bipolar Likert scale, ranging from 1 (*very sad*) to 11 (*very happy*). In the final sample of 61 participants, these scores changed from 7.0 (*SD* = 1.9) before the induction to 2.5 (*SD* = 1.1) after the mood induction.

##### Approach-Avoidance Task (AAT)

As in Study 1, the training was divided into assessment AAT (40 trials) and training AAT (380 trials). The assessment AAT was administered once before and once after the negative mood induction. Both assessments were preceded by 10 practice trials. Immediately after the second assessment and unbeknown to the participants, the training AAT followed. In this phase, contingencies were changed for the training group only, so that now all positive pictures had to be pulled closer and all negative pictures had to be pushed away. The control group received 380 trials of continued assessment (i.e., sham training). Thereafter, and again unbeknown to participants, the training AAT changed into a third assessment AAT which was followed by a booster-training block of 100 trials, in order to ensure that the training effect would not be weakened by the third assessment AAT. The whole joystick task took approximately 30 min, with four breaks in between.

##### Anagram Stress Task

To elicit stress in participants, we again administered the anagram stress task. However, task instructions varied slightly from those in Study 1. For the anagram stress task, 20 letter strings were constructed. Of these, seven were solvable and 13 were not. A Dutch and a German version of the task were created. Participants were instructed on a computer screen to solve as many of these anagrams as accurately as possible, by writing down the correct words on a supplied response sheet. The instructions indicated that most of the anagrams should be easy to solve within the given time. Each anagram was presented individually on the computer screen for 20 s. A clock in the upper right of the screen counted down the seconds to signal for each anagram how much time would be left. The anagram disappeared after twenty seconds, accompanied by a stressful “beep” sound and followed by the next anagram.

#### Procedure

Participants were tested individually. After providing informed consent, participants were randomly assigned to either the positivity training or the (sham training) control group. Then they completed the mood measurements, that is, the SDS and the six mood ratings. Thereafter, participants read the instructions for the following AAT assessment. After completion of this first assessment AAT, the three sequences of the movie “Sophie’s Choice” were presented. Before starting the clips, the experimenter dimmed the light, asked participants to clear their minds of all thoughts and feelings and to put take perspective of the main actress. Following the movie scenes, participants again indicated their current mood state on the six mood ratings. Next, participants received instructions for the succeeding AAT units (second assessment, training, third assessment, booster-training).

In order to assess the effect of the AAT training on emotional vulnerability, participants subsequently completed the anagram stress task. The six mood ratings were administered before and afterwards. Before participants were sent home, they were shown a sequence of the movie “Happy Feet” to elicit a positive mood state (Fitzgerald et al. 2011). To ensure that participants were able to restore their mood in response to the positive mood induction, the six mood ratings were administered for a last time. At the end of the session, participants filled in an awareness check questionnaire. Finally they were paid and given the opportunity to leave behind their e-mail address for later debriefing.

### Results

#### Preliminary Analyses

The group of dysphoric individuals did not significantly differ from the group of non-dysphoric individuals on the compatibility effect prior to the training (dysphoric: *M* = 92, *SD* = 139; non-dysphoric: *M* = 45, *SD* = 99; *t*(59) = 1.55, *p* > .1, *d* = .39). Additionally, one-sample t-tests revealed that in both groups, the compatibility effect was significantly positive (non-dysphoric: *t*(35) = 2.71, *p* = .01, *d* = .92; dysphoric: *t*(24) = 3.30, *p* = .003, *d* = 1.35). Thus, both groups showed an initial positivity bias. A 2 (within-subjects factor *time*: T1 vs. T2) × 2 (between-subjects factor *training*: PT group vs. control group) repeated-measures analysis of variance (ANOVA) revealed no main effects or interactions, neither for the dysphoric nor for the non-dysphoric sample (*p* > .1) indicating that the positivity bias remained unaffected by the mood induction procedure.

#### Approach-Avoidance Training

RTs from the AAT were prepared in the same way as in the first study, for all three assessment phases separately: before the mood induction (T1), before the positivity training (T2) and after the training (T3). Based on the median RTs, the compatibility effects were calculated. These compatibility effects were subjected to a 2 (within-subjects factor *time*: T2 vs. T3) × 2 (between-subjects factor *training*: PT group vs. control group) repeated-measures analysis of covariance (ANCOVA), using the compatibility effect at T1 as covariate. To find out whether the training was effective for both dysphoric and non-dysphoric participants, this analysis was conducted separately for the two groups.

The analysis yielded a significant time × group interaction effect for both dysphoric, *F*(1, 22) = 6.29, *p* = .02, η_p_^2^ = .22, and non-dysphoric participants, *F*(1, 33) = 4.99, *p* = .032, η_p_^2^ = .13. This indicates an increase of the compatibility effects after training in the PT groups (dysphoric: *t*(9) = 5.29, *p* < .001, *d* = 1.67; non-dysphoric: *t*(17) = 2.62, *p* = .018, *d* = .62), compared to the sham-training groups (dysphoric: *t*(14) = 1.13, *p* > .2, *d* = .29; non-dysphoric: *t*(17) = .12, *p* > .9, *d* = .03). Thus, the training was successful in inducing a positivity bias in both dysphoric and non-dysphoric students (see Table 3 for the mean compatibility effects). Moreover, effectiveness of the training did not depend on the size of the initial pre-training bias: Within the PT group, there was no significant correlation of T1 compatibility scores with the T3–T2 change scores, *r*(28) = −.07, *ns.*

**Table 3:** Mean compatibility effects (standard deviations) in milliseconds in Study 2

	Dysphoric		Non-dysphoric	
	Positive training	Control training	Positive training	Control training
Pre-induction	67	131	62	−2
(T1)	(159)	(129)	(84)	(123)
Pre-training	40	127	38	52
(T2)	(86)	(159)	(107)	(93)
Post-training	166	54	171	58
(T3)	(110)	(152)	(226)	(174)

#### Mood After Training

Similar to the first study, immediate effects of training on mood were assessed by means of a MANCOVA, comparing the two groups (training vs. control) on the three mood ratings (happy, sad, content) directly after the training, with the three corresponding pre-training ratings serving as covariates. All MANCOVAs were conducted separately for dysphoric and non-dysphoric individuals. As in the first study, the MANCOVA was favored above repeated-measures ANOVAs because we encountered large violations of sphericity on the stress scales (Stevens 2002). The analysis of the mood ratings yielded no significant difference between training and control group, neither for dysphoric participants, *F*(3, 18) = .39, *p* > .7, η_p_^2^ = .06, nor for non-dysphoric participants, *F*(3, 29) = 1.69, *p* > .1, η_p_^2^ = .15. When including baseline compatibility effect as a covariate, a marginally significant training effect for the non-dysphoric group was revealed, *F*(3, 27) = 2.62, *p* = .071, η_p_^2^ = .23, with participants from the training condition scoring lower on the content subscale than the control group (*M* = 2.94, *SD* = .8 vs. *M* = 3.56, *SD* = .86; *F*(1, 29) = 4.24, *p* = .012, η_p_^2^ = .2). No group differences were found on the remaining subscales (sad: *F*(1, 29) = 2.61, *p* > .1, η_p_^2^ = .06; happy: *F*(1, 29) = .91, *p* > .3, η_p_^2^ = .04) (see Table 4).

**Table 4:** Mean scores (standard deviations) of mood and stress scores before (T2) and after (T3) the training and after (T4) the stress task in Study 2

	Positive training			Control training		
	T2	T3	T4	T2	T3	T4
*Dysphoric*						
Mood						
Happy	1.5	2.7	2.2	1.47	3	2
	(.71)	(1.42)	(1.14)	(.83)	(1.46)	(.93)
Sad	4.3	3	3	4.33	2.6	2.4
	(.68)	(1.05)	(1.56)	(1.23)	(1.18)	(1.55)
Content	2.5	3	1.8	1.93	3.2	1.67
	(1.08)	(1.05)	(.63)	(1.34)	(1.21)	(1.18)
Stress						
Tense	3.2	2.2	2.8	4.07	2.8	4.07
	(1.62)	(1.4)	(1.14)	(1.22)	(.56)	(.88)
Anxious	2.9	1.7	1.8	2.67	2	1.6
	(1.52)	(1.25)	(.99)	(1.63)	(1.41)	(1.24)
Relaxed	2.1	3.2	2.6	1.8	3.27	2.2
	(1.37)	(1.55)	(1.51)	(1.08)	(1.03)	(1.21)
*Nondysphoric*						
Mood						
Happy	1.39	2.83	2.22	1.5	3.06	2.33
	(.98)	(.92)	(1)	(.99)	(1.06)	(1.09)
Sad	4.44	2.39	2.44	4	1.56	1.89
	(1.29)	(1.24)	(1.58)	(1.28)	(1.29)	(1.41)
Content	2.06	2.94	1.39	2.78	3.56	1.5
	(1.47)	(.8)	(1.09)	(1.06)	(.86)	(1.34)
Stress						
Tense	3.89	2.83	3.61	3.11	2.28	3.11
	(.76)	(.92)	(1.42)	(1.28)	(1.78)	(1.68)
Anxious	2.89	1.28	1.06	2.61	1	.94
	(1.45)	(1.02)	(1.06)	(1.38)	(1.33)	(1.26)
Relaxed	1.67	2.72	2.33	2.28	3.67	2.33
	(1.09)	(1.6)	(1.53)	(.9)	(1.78)	(1.33)

#### Stress After Training

The same analyses as above were computed with the three stress ratings (tense, relaxed, anxious). A MANCOVA on the stress scales revealed no training effect for either sample (*p* > .7).^2^

#### Mood After Anagram Task

To investigate effects of the training on emotional reactions to the stressor, a MANCOVA was computed on the mood ratings after the anagram-stress task, with the three mood ratings prior to the anagram task serving as covariates. This analysis revealed no training effect for either sample (*p* > .7).^3^

#### Stress After Anagram Task

For the group of non-dysphoric students, no significant main effect of training on the stress ratings was found, *F*(3, 29) = .95, *p* > .4, η_p_^2^ = .09. For the dysphoric participants, however, the analysis yielded a significant difference in stress ratings between training and control group, *F*(3, 18) = 3.29, *p* = .044, η_p_^2^ = . 35. Univariate ANCOVAs indicated that the training was particularly associated with less tension after the anagram task, *F*(1, 20) = 6.81, *p* = .017, η_p_^2^ = .25, in that participants in the training group showed lower scores on the stress subscale than those in the control group (*M* = 2.8, *SD* = 1.1 vs. *M* = 4.1, *SD* = .9). In contrast, no significant group differences were found on the other two subscales (anxious: *F*(1, 20) = 1.05, *p* > .3, η_p_^2^ = .05; relaxed: *F*(1, 20) = .25, *p* > .6, η_p_^2^ = .01). These results show that dysphoric participants in the training group were less tense than dysphoric individuals in the control group. The training did not have this protective effect for non-dysphoric students (see Table 4). When adding baseline compatibility scores as covariate, the main effect of training on tension in the dysphoric group became non-significant, *F*(1, 18) = 1.62, *p* > .2, η_p_^2^ = .08, while the interaction of training with initial compatibility effect reached marginal significance, *F*(1, 18) = 3.04, *p* = .098, η_p_^2^ = .15. This suggests that the size of the initial compatibility bias moderates the training effect, with larger bias scores being related to less tension after the training.

To further investigate whether the training was indeed significantly more effective in decreasing tension in dysphoric than in non-dysphoric participants as indicated by the ANCOVA above, an additional 2 (*training*: positivity vs. control) × 2 (*SDS group*: dysphoric vs. non-dysphoric) ANCOVA was conducted on the tension ratings after the anagram task, again using the tension ratings prior to the task as covariate. Results revealed a marginally significant interaction effect of training with SDS group, *F*(1, 56) = 3.33, *p* = .073, η_p_^2^ = .06. Although this interaction effect fell short of statistical significance, it is compatible with our finding that the training differentially affected emotional vulnerability to stress in dysphoric and non-dysphoric individuals. Finally, we also computed a comparable analysis in which SDS scores were used as a continuous variable, instead of using them to create two groups of dysphoric vs. non-dysphoric students. In this analysis, the critical SDS × training group interaction was not significant, *F*(1, 56) = .908, *p* > .3, η_p_^2^ = .02. This suggests that the relation between dysphoria and training benefit may be non-linear, supporting the cut-off score suggested by Zung (1973).

### Discussion

The primary aim of the second study was to investigate whether a general positivity training is able to induce a bias towards positive materials, compared to a neutral control training. We investigated this question in a sample of dysphoric and non-dysphoric students who received a negative mood induction. Unfortunately, a substantial number of participants had to be excluded from the analyses because for them, the mood induction was ineffective (about 40 % of the total sample). This surprisingly low potency of the mood induction limits our findings, as well as the resulting small sample size of the four experimental participant groups. In line with our expectations, we successfully modified a positivity bias to emotional information by means of the training. Participants who received the positivity training showed an increase in their compatibility effect after the training, and thus a stronger positivity bias. This was true despite the fact that both dysphoric and non-dysphoric participants showed a positive bias before the training already. No such change was found in the control group. Similar to the first study, the training did not directly influence participants’ mood, neither in the group of dysphorics nor in the group of non-dysphoric students. These results are in line with the meta-analytical review by Hallion and Ruscio (2011), which suggests that CBM trainings reveal their effects only after exposure to a stressor.

The second aim was to investigate if stress reactivity was affected by the general positivity training, which would be indicative of a therapeutic value. In line with our hypothesis, we found that the training served to modify emotional vulnerability and as a consequence, functioned as a buffer against stress. The PT group showed lower tension levels in response to the anagram task than the control group. In this regard, the effectiveness of the general positivity training is comparable to former CBM approaches, despite the unspecific selection of generally positive and negative stimulus materials. However, the findings have to be interpreted cautiously considering the fact that we relied on self-reported stress. The two ratings “tension” and “relaxation” can be interpreted as two counterparts of the physiological stress reaction, while the rating “anxiety” can be seen as the emotional consequence of this physiological reaction. In our study, only tension reduced significantly, while this buffer effect of training on emotional vulnerability was not present in the sample of non-dysphoric students. This seems to contradict earlier studies demonstrating effects in unselected samples of students (MacLeod et al. 2002; See et al. 2009). A possible explanation might be that the anagram stress task was of relatively low intensity. The induced stress may have been just strong enough to provoke tension in dysphoric individuals, but may have been too mild to produce the equivalent emotional reaction, that is, anxiety in dysphoric students. Furthermore, non-dysphoric individuals may have been even less emotionally affected. This explanation is conceivable because, in contrast to the anagram stress task used by MacLeod et al. (2002), our participants were not videotaped, and they did not receive negative feedback about their performance in this task. As in Study 1, the intensity of the stressor in the present study might still have been too low for the group of non-dysphoric participants to elicit meaningful variations in stress responses between the training and the control condition. For follow-up studies focusing on emotional vulnerability, we therefore recommend to ensure enough variation in the experienced levels of stress. Future studies might also benefit from focusing on disorder-relevant situations, for example self-esteem-related situations in depressive patients. Above that, stress measurements that do not rely solely on self-report (i.e., physiological measures of stress) should be included in future studies.

In sum, the present findings are in line with earlier research showing that cognitive biases can be induced without having an immediate consequence on the emotional state of a person. Thus, the general positivity training does not serve as a positive mood induction that would help to recover from negative emotions. Rather, as already suggested by Mathews and MacLeod (2005), emotional consequences become apparent in subsequent situations, when the induced bias is actively used to process emotional information, such as in the anagram stress task.

## General Discussion

Both our studies demonstrate that it is possible to modify approach-avoidance tendencies by means of a general CBM training that, in contrast to existing CBM methods, relies on a disorder-non-specific selection of positive and negative stimuli. This suggests that CBM training effects are not restricted to the use of content-specific information, but extend to the modified processing of emotionally valenced information in general. In the first study, we showed that the negativity training can reverse an initial positivity bias in a sample of healthy individuals. However, the positivity training was not able to further strengthen this initial bias. In order to obviate this potential ceiling effect, the second study focused on both an emotionally vulnerable group and a healthy group in a sad mood state. In this second study, we successfully modified a bias in both groups, which demonstrates that the positivity training was indeed able to strengthen an existing positivity bias. In this respect, the results of Study 2 were inconsistent with those of Study 1, and additional research is needed to reconcile these discrepant findings.

Compared to most other CBM training procedures, this general AAT is unique in that it targets emotion-driven action tendencies. Approach-avoidance models (e.g., Elliot 2006) state that approach motivations are triggered by positive stimuli, whereas avoidance motivations are triggered by negative stimuli. In line with this, we developed a training that made use of generally positive and negative stimuli in order to strengthen the corresponding action tendencies. As demonstrated in the first study, the modification of participants’ approach-avoidance tendencies transferred to attentional processing. This supports the assumption that approach-avoidance movements influence the motivational orientation and subsequently enhance the processing of positive or negative stimuli (Neumann and Strack 2000). Hence, the effects of the general AAT are not merely limited to the modification of action tendencies. They translate to the processing of emotionally valenced stimuli in general, emphasizing the encompassing nature of this CBM training. It is unfortunate that time constraints did not allow us to include the dot probe task in Study 2, so these results will have to be replicated in future studies.

As expected, the training had no immediate effects on state emotion, neither for dysphoric nor for non-dysphoric students. This finding is in line with earlier studies, showing that biases in cognitive processing can be induced without eliciting any direct emotional effects (MacLeod et al. 2002; Mathews and MacLeod 2005). Results of both studies indicate that the general AAT does not work as a mood induction in itself. Instead, the mediating role of environmental factors, as for instance the presence of a stressful situation, appears to be crucial when it comes to effects of the training on mood, as indicated by the attenuated stress reactivity of the dysphoric individuals in response to the anagram stress task.

It should be kept in mind that the dysphoric group in Study 2 did not show a negativity bias before training. Therefore, our conclusions regarding the effectiveness of the general positivity training in attenuating stress-reactivity are based on the fact that we were able to strengthen a pre-existing positivity bias. However, we did not reverse an initial negativity bias into a positive one. Individuals with emotional disorders usually do show a negativity bias (Mathews and MacLeod 2005). Consequently, future research should investigate whether the reversal of an initial negativity bias into a positive one is possible.

The findings in the second study nicely complement earlier findings, for instance by MacLeod et al. (2002). Only a few studies, however, have focused on the role of positive informational processing in emotional vulnerability and dysfunction so far. In line with Taylor et al. (2011), our study provides encouraging support for the notion that enhancing a bias associated with a healthier processing of emotional information serves to attenuate stress-reactivity, in our case in emotionally vulnerable individuals (i.e., dysphoric students). Complementing the study by Taylor and colleagues in anxious participants, this study extends previous encouraging results of a positive CBM training to a new group.

To our knowledge, this is the first study indicating that a general positivity training, which is based on the modification of action tendencies, changes stress reactivity and thus might be of therapeutic value in individuals commonly known to lack the positive processing biases that are found in healthy individuals. It is also the first showing cross over effects with attentional biases. A necessary subsequent step would be to investigate whether the induction of a positivity bias also serves to decrease emotional vulnerability in a sample of clinically depressed patients. Given that participants in the second study showed only mild symptoms of depression, it would be premature to draw conclusions regarding the effectiveness of the training in clinical samples, especially because our group of dysphoric students showed a surprising positivity bias already before training.

Although we did not find any effects of the training on measures of mood directly after the training, the general positivity training might still be capable of reducing depressed feelings in the long term. It is conceivable that promoting healthy processing of emotional information might serve to decrease depressed mood. However, it might only do so over a prolonged period of time, when the modified bias has been repeatedly deployed to selectively process positive over negative emotional information. In this way, a strengthened positivity bias might not only reduce negative reactions to adverse situations, but might similarly allow people to benefit more from positive experiences. They might then show a greater increase of mood in response to a positive situation, compared to those with a weaker positivity bias or even a bias towards negative materials.

Finally, it remains unclear which training component (pulling positive pictures or pushing negative pictures) is the effective ingredient when it comes to modifying pre-existing approach-avoidance tendencies and subsequently to attenuate stress reactivity. The effect of the training might be due to the “avoid negative” component, to the “approach positive” component, or due to a combination of both. Future research should focus on identifying the crucial components that make the training work or which might make it even more effective (e.g., by using only positive stimuli that have to be approached continuously). Research should also investigate the temporal stability of an induced positivity bias. This is particularly important when considering the use of such training as an additional treatment instrument in clinical settings (e.g., Asnaani et al. 2014). In disorders such as depression, clinical improvement might only be achieved by an enduring change in cognitive biases. For that purpose, follow-up studies are recommended which focus on more extensive training procedures, with a greater number of sessions spread over a prolonged period of time.

## Footnotes

1 Including the baseline compatibility effect as a covariate did not change the results (*p* > .162).

2 These results remained non-significant after including the baseline compatibility effect as a covariate (*p* > .791).

3 Including the baseline compatibility effect as a covariate did not change these results (*p* > .475).
